# Calcium Channel-Dependent Molecular Maturation of Photoreceptor Synapses

**DOI:** 10.1371/journal.pone.0063853

**Published:** 2013-05-13

**Authors:** Nawal Zabouri, Silke Haverkamp

**Affiliations:** Neuroanatomy, Max-Planck-Institute for Brain Research, Frankfurt am Main, Germany; Virginia Tech Carilion Research Institute, United States of America

## Abstract

Several studies have shown the importance of calcium channels in the development and/or maturation of synapses. The Ca_V_1.4(α_1F_) knockout mouse is a unique model to study the role of calcium channels in photoreceptor synapse formation. It features abnormal ribbon synapses and aberrant cone morphology. We investigated the expression and targeting of several key elements of ribbon synapses and analyzed the cone morphology in the Ca_V_1.4(α_1F_) knockout retina. Our data demonstrate that most abnormalities occur after eye opening. Indeed, scaffolding proteins such as Bassoon and RIM2 are properly targeted at first, but their expression and localization are not maintained in adulthood. This indicates that either calcium or the Ca_V_1.4 channel, or both are necessary for the maintenance of their normal expression and distribution in photoreceptors. Other proteins, such as Veli3 and PSD-95, also display abnormal expression in rods prior to eye opening. Conversely, vesicle related proteins appear normal. Our data demonstrate that the Ca_V_1.4 channel is important for maintaining scaffolding proteins in the ribbon synapse but less vital for proteins related to vesicular release. This study also confirms that in adult retinae, cones show developmental features such as sprouting and synaptogenesis. Overall we present evidence that in the absence of the Ca_V_1.4 channel, photoreceptor synapses remain immature and are unable to stabilize.

## Introduction

At the first retinal synapse, photoreceptors relay light-evoked signals to horizontal and bipolar cells. To effectively convey their signal and sustain their activity, primary sensory neurons such as photoreceptors and hair cells require a particular type of chemical synapse, known as ribbon synapse. In these structures, a large array of proteins is organised around an electron dense synaptic ribbon. L-type voltage-dependent calcium channels (L-VDCC) are vital for transmission at the photoreceptor terminal, as they allow the Ca^2+^ influx that initiates exocytosis (see for recent review [1]). Immunohistochemical data show that the channel Ca_V_1.4(α_1F_) is associated with the active zone at the base of the ribbon in photoreceptors [2], [3]. Another channel Ca_V_1.3(α_1D_), containing a different isoform of the pore forming α1-subunit, is mainly expressed in hair cell ribbon synapses, but also in photoreceptors [4], [5]. However, while removal of Ca_V_1.3(α_1D_) profoundly affects hearing, it does not alter retinal responses [6]–[9]. Conversely, elimination of Ca_V_1.4(α_1F_) strongly impairs retinal function [10], [11]. Interestingly, a recent study revealed that calcium influx through Ca_V_1.3(α_1D_) regulates ribbon size during development and contributes to the refinement and maintenance of synaptic contacts in hair cells [12].

In the retina, several lines of evidence demonstrate that partial or complete interference with Ca_V_1.4(α_1F_) expression cause congenital stationary night blindness (CSNB2) in humans and a diminished or abolished ERG b-wave in mice [6], [8], [11], [13]. The *Cacna1f^nob2^*(*nob2*) mouse is a spontaneous CSNB2 model that expresses about 10% of Ca_V_1.4(α_1F_) transcripts [14]. This model shows no b-wave but mostly normal photopic optokinetic acuity [11], [15], while the Ca_V_1.4(α_1F_) knockout (Ca_V_1.4(α_1F_)-KO) mouse exhibits neither [8], [15]. Anatomically, both Ca_V_1.4(α_1F_)-KO and *nob2* retinae display untethered ribbons and several anomalies in the photoreceptors’ presynaptic protein distribution as well as outgrowth of rod bipolar and horizontal cell processes into the outer retina [10], [16]. In addition to these changes, cones display an abnormal morphology and degenerate in aged Ca_V_1.4(α_1F_)-KO [17].

The sequence of events leading to the formation of a photoreceptor ribbon synapse in mouse was studied in detail [18], yet the elements involved in the maturation of this synapse remain unknown. In cultured photoreceptors, Ca_V_1.4(α_1F_) is required for structural plasticity in rods [2].

Activity-dependent Ca^2+^ influx into the synapse accounts for a very large proportion of the photoreceptor calcium currents [19], thus Ca_V_1.4(α_1F_) is a crucial provider of Ca^2+^ in photoreceptors. In addition to its role in synaptic transmission, Ca^2+^ also acts as an intracellular second messenger and plays important roles both in adulthood and during development. In particular, Ca^2+^ influx through L-VDCC is implicated in several developmental processes. For instance, it can be involved in neuronal differentiation [20] and neurite outgrowth [21] as well as in synapse maturation and stabilization [12]. Ca^2+^ can also affect signaling pathways leading to transcriptional activation and, ultimately, to changes in gene expression involved in neuronal survival and plasticity [2], [22], [23].

Given the demonstrated role of Ca_V_1.3(α_1D_) in the synaptic maturation of hair cells, we investigated the involvement of Ca_V_1.4(α_1F_) in the maturation of photoreceptor ribbon synapses. The Ca_V_1.4(α_1F_) knockout shows abnormal ribbons both in adults and in pups [17], but the extent of the synaptic defects remains unknown. Thus, we dissected the timeline of molecular identity loss in the photoreceptor ribbon synapse. We analyzed the expression of several presynaptic proteins during maturation of the ribbon synapse and in adulthood. Furthermore, the direct association between the abnormal cone morphology found in aged Ca_V_1.4(α_1F_)-KO and neurodegeneration has yet to be confirmed. Therefore we studied the cone morphology at different ages in the Ca_V_1.4(α_1F_)-KO. Our data demonstrate that the absence of the Ca_V_1.4(α_1F_) channel strongly affects the maintenance of scaffolding elements in the synapse, but not the vesicle-related machinery. In addition, cones start to remodel several months prior to the onset of degeneration, suggesting two separate mechanisms. In the absence of Ca_V_1.4(α_1F_), cones retain their ability to seek and establish new synapses even in the adult, suggesting activity and/or Ca_V_1.4(α_1F_) are necessary for cones to become fully mature.

## Materials and Methods

### Animals and Tissue Preparation

*CACNA1F^tm1.1Sdie^* (Ca_V_1.4(α_1F_)-KO) mice were obtained from Dr. Marion Maw [10], bred in-house and maintained on a 12-hours light/dark cycle. All animal procedures were carried out in accordance with institutional guidelines of the Max Planck Institute for Brain Research (Frankfurt) and following the standards described by the German animal protection law (Tierschutzgesetz). Wild type (WT) and Ca_V_1.4(α_1F_)-KO C57BL6 mice were deeply anesthetized with isoflurane and sacrificed by decapitation, at various ages ranging from postnatal day 9 to 10 months (2 to 3 animals per time point). The eyes were quickly removed and immersed in phosphate-buffered 4% paraformaldehyde (PFA). The anterior segments were removed and the posterior eyecups were fixed for 15–30 minutes in PFA. The eyecups were then cryoprotected in 30% sucrose, the retinae isolated, frozen in Jung II mounting media, cut in vertical sections (14–20 µm) with a cryostat (Leica Microsystems, Germany), collected on electrostatic slides, and stored at –20°C until use. In order to evaluate the extent of cone degeneration, some mature retinae were immunolabeled as flat mounts.

### Immunohistochemistry

Immunolabeling was performed using the indirect fluorescence method. Sections were incubated overnight in a mixture of primary antibodies (see table 1 for complete list and references) at the appropriate concentrations in a blocking solution containing 3% normal donkey or horse serum, 0.5% Triton X-100 in phosphate-buffered saline (PBS - phosphate 0.1 M, 0.9% NaCl, pH 7.4). The following day, the sections were incubated with the appropriate secondary antibodies in blocking solution for 1 hour, and then mounted with AquaPoly/mount (Polysciences, Eppelheim, Germany). For cone arrestin immunolabeling, an antigen retrieval protocol was used. Briefly, the slides were first incubated in 0.6% CaCl_2_ solution at 37°C, then in citric buffer (0.01 M, pH 6) for 20 min at 80°C. Then immunolabeling was carried out as above.

**Table 1:** Antibodies.

Primary antibodies					
Protein	Host	Dilution	Immunogen		Company
CtBP2	Rabbit	1∶10000	Synthetic peptide (aa 431–445 of rat CtBP2).		Synaptic Systems, Goettingen, Germany.
	Mouse	1∶5000	C-terminal mouse CtBP2 (aa 361–445).		BD transduction, Heidelberg, Germany.
Bassoon	Mouse	1∶1000	Recombinant rat Bassoon protein (aa 756–1001).		StressGen Biotechnology, San Diego, CA, USA.
Veli3	Rabbit	1∶5000	C-terminus of the rat Velis (aa 182–197).		Zymed (Invitrogen).
PSD-95	Mouse	1∶200	Purified recombinant rat PSD-95.		ABR–Affinity bioReagents,Golden, CO, USA.
vGluT1	Guinea-pig	1∶10000	Synthetic peptide (aa 542–560 of rat vGluT1).		Chemicon, Temecula, CA, USA.
VAMP2	Rabbit	1∶100	Synthetic peptide (aa 1–18 of Rat VAMP2).		Abcam, Cambridge, UK.
RIM2	Mouse	1∶100	Recombinant protein (aa 461–987) of rat RIM2.		Synaptic Systems
Complexin 4	Rabbit	1∶20000	Recombinant full length of mouse complexin 4.		Synaptic Systems
Complexin 3	Rabbit	1∶10000	Recombinant full length of mouse complexin 3		Synaptic Systems
Synaptophysin	Mouse	1∶500	Synaptosome preparation from rat retina		Sigma-Aldrich.
S-opsin	Goat	1∶100	Peptide corresponding to sequence within aa1–50 of human blue-sensitive opsin.		Santa Cruz Biotechnology, Santa Cruz, CA, USA.
Calbindin	Rabbit	1∶2000	Recombinant rat calbindin D-28k		Swant, Bellinzona, Switzerland.
PKC	Mouse	1∶100	PKC purified from bovine brain.		BioDesign, Saco, ME, USA
Green opsin	Rabbit	1∶1000	Synthetic peptide (aa 340–363 of human opsin)		Santa Cruz Biotechnology, Heidelberg, Germany.
Glypho	Rabbit	1∶2000	aa 826–841 of the rat muscle-specific sequence		Gift from Dr. Hamprecht [24].
PNA	NA	1∶1000	NA		Invitrogen, Darmstadt, Germany.
mCar	Rabbit	1∶10000	N-terminal of human cone arrestin (aa 98–388).		Gift from Dr. Baehr [25].
**Secondary antibodies**					
Anti-mouse	Donkey	1∶500		Jackson ImmunoResearch	
Anti-rabbit					
Anti-goat					
Anti-mouse	Donkey	1∶500		Mobitec	
Anti-rabbit					
Anti-goat					

### Antibody Characterization

The antibodies against the C-terminal binding protein 2 (CtBP2), a RIBEYE homologue, recognize ribbons in mammalian retina and produce a distinctive immunoreactivity pattern of horseshoe-shaped synaptic ribbons in the OPL and dense puncta in the IPL. The immunolabeling we observed in WT mice was similar to that reported in previous studies using these and alternative antibodies. Both antibodies were further characterized by western blot on retinal extracts and both reacted with a 110 and a 120 kDa band (manufacturer's technical information, [26]).

Bassoon is a well-established marker for photoreceptor ribbon synapses; it also produces a horseshoe-shaped synaptic pattern in the OPL and dense puncta in the IPL. The expression pattern presented here is similar to that previously described by us and others. The specificity of this mouse anti-Bassoon (StressGen Biotechnology) was tested by western blot on retinal extracts and it reacted with a single band at 420 kDa (manufacturer's technical information). Furthermore, this antibody did not produce any labeling in Bassoon knockout retinae [27], [28].

Veli3 is a scaffolding protein expressed at the photoreceptor synaptic membrane and the immunolabeling we obtained in this study is similar to that presented elsewhere [10], [29]. The specificity of the antibody was tested by western blot and it was shown to react with a single band at 22 kDa (manufacturer's technical information, [29]). Furthermore, this antibody detects only Veli3 upon heterologous expression of Rho-1D4-tagged Veli isoforms in 293-EBNA cells [29].

PSD-95 is a well-established marker for photoreceptor terminals in mammalian retina [30]. The mouse anti-PSD-95 (ABR Affinity BioReagents) reacts with a major double bond at about 95 kDa (manufacturer's technical information).

The vesicular glutamate transporter 1 (vGluT1) labels synaptic terminals of photoreceptors and bipolar cells. Its distribution is highly conserved across mammalian species [31]. The guinea-pig anti-vGluT1 (Chemicon) reacts with a single band at 62 kDa (manufacturer's technical information). The expression pattern obtained here was consistent with other reports [31].

Synaptobrevin 2 (VAMP2) is a synaptic vesicle-associated v-SNARE protein and is expressed in photoreceptor synapses [32]. The rabbit anti-VAMP2 (Abcam) reacts with a single 19 kDa band (manufacturer's technical information). The expression pattern obtained here was consistent with previous reports [32].

Rab 3 interacting molecule 2 (RIM2) labels the arciform density compartment of the photoreceptor ribbon complex [26], [33]. The rabbit anti-RIM2 (SYSY) was tested by western blot and it reacts with a major band at about 180 kDa (manufacturer's technical information, [34]).

Complexin (Cplx) 3 and 4 are cytosolic proteins responsible for binding and stabilizing assembled SNARE complexes. Cplx3 is strongly expressed in cones and lightly immunoreactive in rods, while cplx4 is only expressed in rods [35], [36]. Both antibodies were tested on their respective knockout models and neither showed any immunoreactivity [37].

Synaptophysin is a vesicular protein. Mouse anti-synaptophysin (Sigma) was tested by western blot and it detected a single band of appropriate size at 38 kDa (manufacturer's technical information). The pattern of immunoreactivity presented in this study is similar to that described elsewhere [38].

The S-opsin antiserum (Santa Cruz) was tested by western blot and it recognized a major band at about 40 kDa [39]. This antibody stained S-cones in mouse [40], [41], rat [42], and primate retinae [43]. The expression pattern we present is similar to that in other reports [40], [41].

Glycogen phosphorylase is expressed in cone photoreceptors. The antibody we used was characterized by western blot and it reacted with a single band at 97 kDa [24]. This antibody produced the same pattern of immunolabeling as previously reported [40].

Cone arrestin (mCar) is a well-established cone marker. This antibody was characterized by western blot and reacts with a major band at about 50 kDa [25]. It produces the same expression pattern as obtained with other cone arrestin antibodies [40].

Calbindin is a well-known marker of horizontal cells and subpopulations of amacrine cells. This calbindin antibody (Swant) detects 28-kDa calbindin on western blots and cross-reacts slightly with calretinin (manufacturer's technical information). The immunolabeling presented in this study is consistent with other reports [44].

Protein Kinase C_α_ (PKC) is specifically expressed in rod bipolar cells and dopaminergic amacrine cells [45]. The PKC antibody recognizes the purified PKC on Western blots and specifically immunoprecipitates PKC from cell lysates of 328 glioma and SVK14 cell lines [46]. This antibody reacts with PKC-α/β-1/β-2 isoforms (manufacturer's data sheet).

### Confocal Microscopy and Image Analysis

Samples were imaged with a confocal microscope (Olympus Fluoview FV1000 or Zeiss LSM5 Pascal) equipped with Helium-Neon and Argon lasers. Brightness and contrast of the final images were adjusted using Adobe Photoshop CS5.

The density of Bassoon (Bsn) puncta at ribbon synapses was manually counted through stacks (70×70×6 µm) at postnatal day (P) 13 and P30 in Ca_V_1.4(α_1F_)-KO mice. Given that Bassoon is also expressed at conventional synapses below the photoreceptor terminals, we counted only Basson puncta within photoreceptor terminals, identified by vGluT1 immunoreactivity. A total of 9 fields (4 animals) were analysed and the averaged density was calculated per time point. Of the total Bsn puncta, we calculated the percentage of puncta that were co-localized with CtBP2 (ribbon marker). The averaged Bsn density and percentage of co-localization with CtBP2 at P13 and P30 were compared by independent student t-tests.

### Transmission Electron Microscopy

The eyes were quickly removed, immersed in PFA, the retinae were isolated and cut into small pieces. The retinal pieces were then fixed in 2.5% glutaraldehyde in 0.1 M cacodylate buffer (pH 7.4) for two hours, dehydrated in 30% sucrose solution in 0.1 M cacodylate buffer, post-fixed in osmium tetroxide (1% w/v in 0.1 M cacodylate buffer) for 1 h, and pre-stained with 2% uranyl acetate in acetone for 30 minutes. The specimens were then dehydrated in a graded series of acetone solutions and embedded in Epon (Serva, Heidelberg, Germany). A series of ultrathin sections (50 nm) were collected on copper grids and contrasted with uranyl acetate and lead citrate. The specimens were examined with a Zeiss Leo912 AB Omega transmission electron microscope (Carl Zeiss SMT AG, Oberkochen, Germany) and photographed with a wide-angle Dual Speed 2K-CCD camera in combination with ImageSP software (TRS, Moorenweis, Germany). Images were adjusted as described above.

## Results

**Figure 1:**
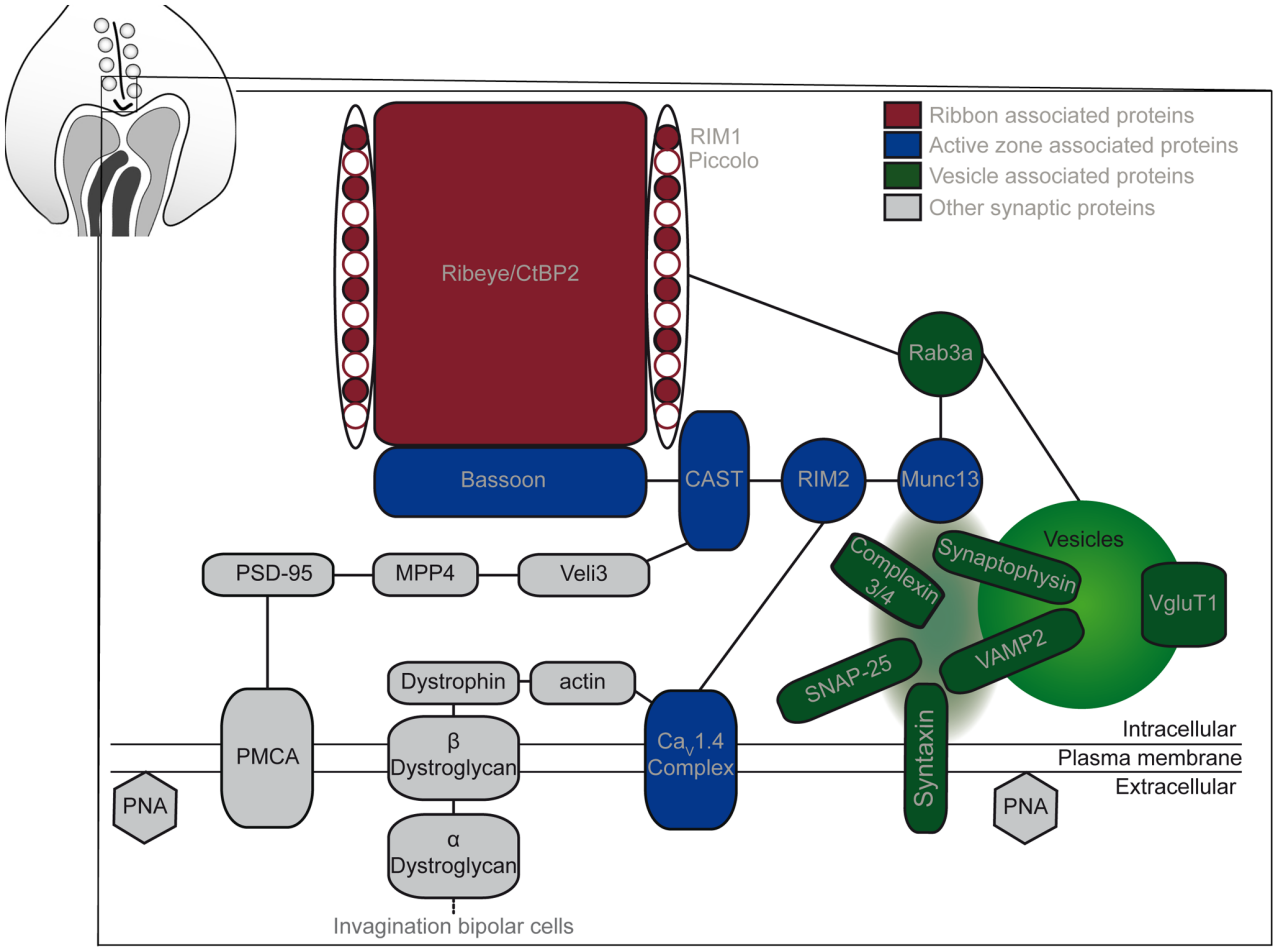
Synaptic proteins investigated in the Ca_V_1.4(α_1F_)-KO. In photoreceptor synapses, proteins are divided in several compartments: the ribbon associated proteins are shown in red, the active zone associated proteins in blue, and vesicle associated proteins in green. Additional proteins are important for synaptic functions and some of these proteins are presented in grey.

Photoreceptor synaptic proteins have been studied extensively and divided into several complexes. tom Dieck et al [26] divided known proteins of the cytomatrix at the active zone in two molecular compartments: a ribbon-associated and an active zone associated complex. The ribbon-associated proteins are more closely associated with the ribbons and are untethered in the Bassoon knockout mice [26]. Ribbon-associated proteins, active zone associated proteins and vesicle associated proteins are the three groups of synaptic proteins we have investigated in the Ca_V_1.4(α_1F_)-KO for this study. Figure 1 illustrates the proteins we have investigated as well as additional ones investigated by other groups in similar models.

The first part of the study focuses on the synaptic structure in photoreceptors of the Ca_V_1.4(α_1F_)-KO retina, while the second section explores cone plasticity in this model.

### Retracting Rod Synapses in the Ca_V_1.4(α_1F_)-KO

**Figure 2:**
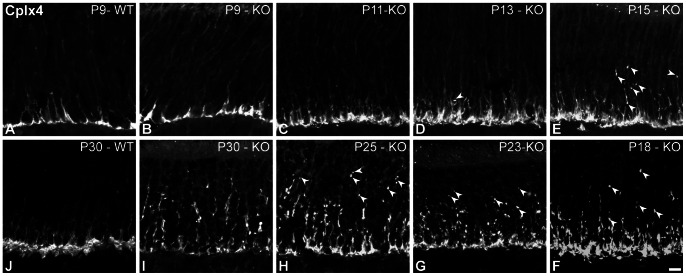
Distribution of photoreceptor terminals at different ages in the Ca_V_1.4(α_1F_)-KO. Photoreceptor terminals are visualized with complexin 4 (Cplx4). **A–J**: Vertical sections from P9-WT (**A**), P9-KO (**B**), P11-KO (**C**), P13-KO (**D**), P15-KO (**E**), P18-KO (**F**), P23-KO (**G**), P25-KO (**H**), P30-KO (**I**) and P30-WT (**J**) mouse retinae. At all ages, ectopic terminals are distinguishable as indicated with white arrowheads. Scale bar = 5 µm.

Complexin 4 (Cplx4) is one of the few proteins involved in vesicle exocytosis that is also established as a specific marker for photoreceptor ribbon synapses (see for review [1]). Cplx4 is expressed in rod synapses [36] and is responsible for binding and stabilizing assembled SNARE complexes during Ca^2+^-triggered fusion of synaptic vesicles [36], [37]. The expression of this protein was preserved in rod synapses in the Ca_V_1.4(α_1F_)-KO at all investigated ages. Therefore, we used it to study photoreceptor terminal position over time in the Ca_V_1.4(α_1F_)-KO (figure 2). At P9 (figure 2A–B), rod synapse localization was undistinguishable between WT and Ca_V_1.4(α_1F_)-KO. This remained the case until eye opening. By P13 (figure 2D), a few ectopic synapses appeared in the outer nuclear layer (ONL - arrowheads) in the Ca_V_1.4(α_1F_)-KO. The number of ectopic synapses progressively increased over the following weeks (figure 2E–I), as more and more Cplx4 immunolabeled terminals appeared in the ONL.

**Figure 3:**
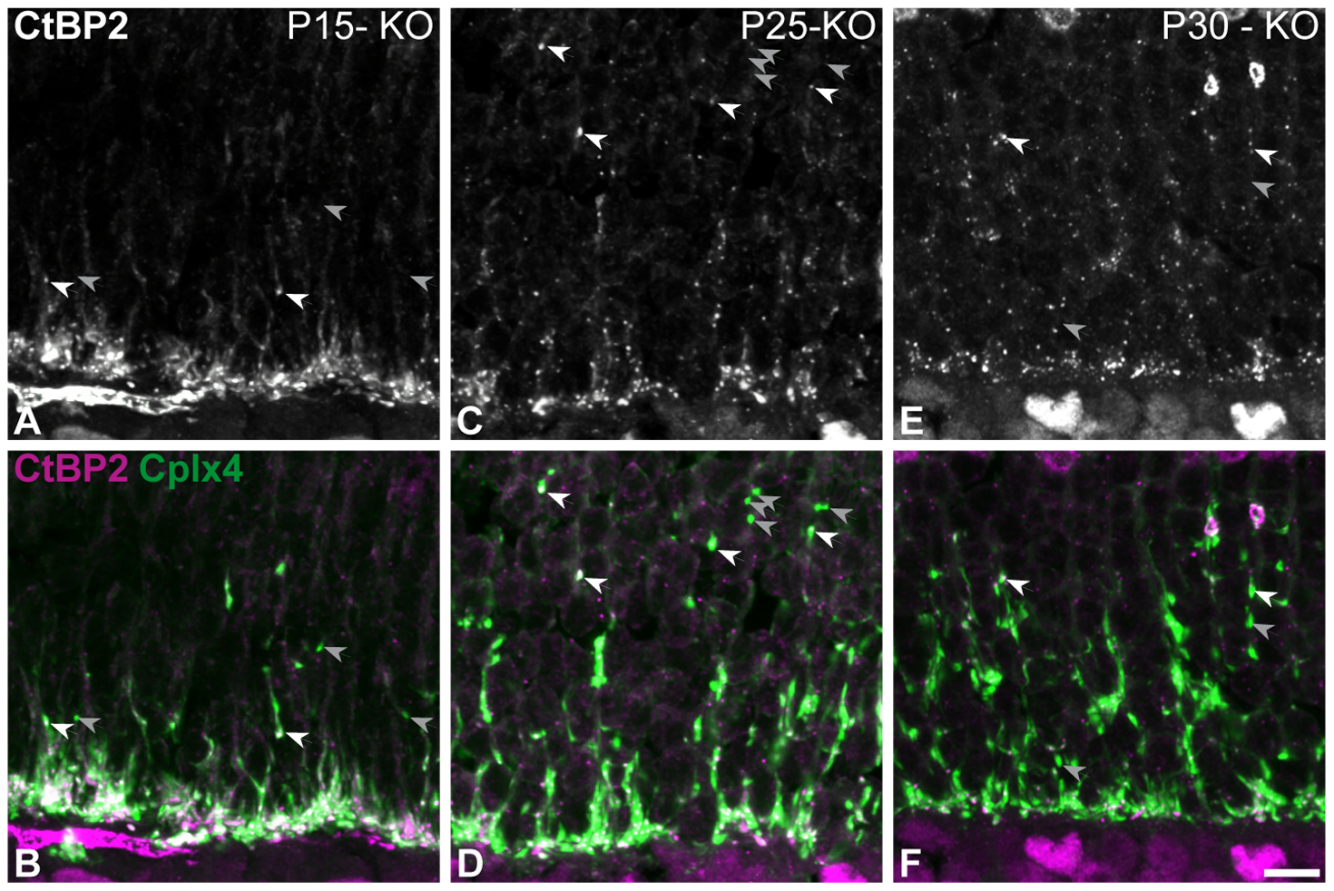
Some isolated terminals are new terminals in Ca_V_1.4(α_1F_)-KO retinae. (**A–F**) Vertical sections from P15 (**A, B**), P25 (**C, D**) and P30 (**E–F**) mouse Ca_V_1.4(α_1F_)-KO retinae. (**A–F**) Confocal micrographs of retinae co-immunolabeled for complexin 4 (Cplx4) and C-terminal binding protein 2 (CtBP2). CtBP2 is presented alone in grayscale in the top row, the CtBP2 (magenta) and Cplx4 (green) signals are presented merged in the bottom row. Potentially quickly retracting isolated terminals that are Cplx4-positive/CtBP2-positive are indicated by white arrows, new or degenerating terminals that are Cplx4-positive/CtBP2-negative are indicated by grey arrows (see text for explanation). Scale bar = 5 µm.

Next we co-labeled CtBP2 (a ribbon marker) with Cplx4 (figure 3). The expression of CtBP2 is presented separately in the top row of figure 3 and merged with Cplx4 in the bottom row. In line with other publications on this model, CtBP2 expression was maintained in the Ca_V_1.4(α_1F_)-KO retina. It did not, however, adopt the normal horseshoe-shape it had in WT retinae [10]. In most synapses Cplx4 and CtBP2 were co-expressed (white arrows). However, we also found examples of Cplx4-positive/CtBP2-negative synapses (gray arrows). These few terminals could either be remnants of degenerating synapses or a few newly established synapses, as ribbons have been shown to appear late in new ectopic synapses [47], [48] and rod photoreceptors are known to be very plastic in synaptopathic models [48], [49]. Moreover, in agreement with published evidence of floating ribbons in similar models [16], [17], we observed numerous CtBP2 puncta that were not co-localized with Cplx4. Given that the calcium channel Ca_V_1.4(α_1F_) is part of the active zone compartment, we set out to evaluate whether the latter was disorganized.

### Active Zone Associated Proteins in the Ca_V_1.4(α_1F_)-KO Mice

**Figure 4:**
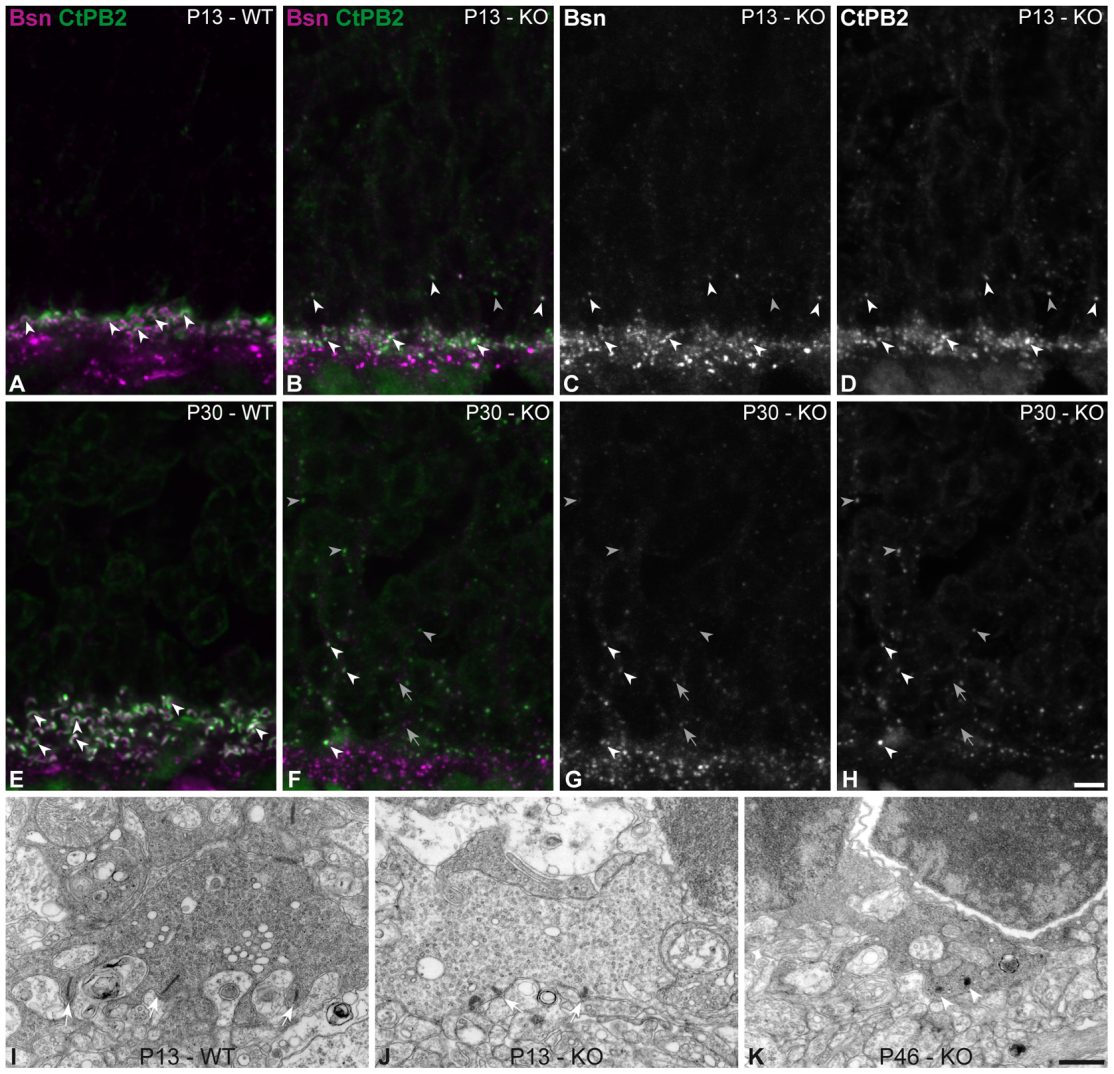
Association of Ribeye and Bassoon at different ages. (**A–H**) Vertical sections from P13 WT (**A**) and Ca_V_1.4(α_1F_)-KO (**B–D**), and P30 WT (**E**) and Ca_V_1.4(α_1F_)-KO (**F–H**) retinae. Confocal micrographs of retinae co-immunolabeled for Bassoon (Bsn - magenta) and CtPB2 (green) are merged in **A–B** (P13) and **E–F** (P30); in addition each staining is presented separately in grayscale: **C and G** depict Bassoon staining; **D and H** show CtBP2 distribution. White arrowheads indicate the terminals where CtBP2 and Bassoon are associated, grey arrowheads indicate isolated Bassoon in the ONL, and grey arrows indicate isolated CtBP2 in the ONL. Scale bar = 5 µm. **I–K:** Electron micrographs of cone pedicles in WT and CaV1.4(α1F)-KO at different ages. (**I**) Cone pedicle containing three presynaptic ribbons in a P13 WT mouse. (**J**) Cone pedicle containing two presynaptic ribbons in a P13 Ca_V_1.4(α1F)-KO. (**K**) Synaptic terminal containing two spherical presynaptic ribbons (white arrowheads) in an adult Ca_V_1.4(α_1F_)-KO. Scale bar = 0.5 µm.

Bassoon is a scaffolding protein involved in anchoring the ribbon to the membrane in photoreceptor terminals [50], thus the next step was to analyze the expression of Bassoon in the Ca_V_1.4(α_1F_)-KO mice. Therefore, we co-labeled Bassoon and CtBP2 (figure 4). At all analyzed ages (P13– figure 4B, C and P30– figure 4E, G), Bassoon was present in the outer plexiform layer (OPL), as well as in some ectopic synapses in the ONL of Ca_V_1.4(α_1F_)-KO retinae. In line with other published evidence, Bassoon was present in the OPL at ribbon synapses of the photoreceptors and at conventional synapses below the photoreceptor terminals [27]. The overall number of Bassoon puncta at photoreceptor synapses declined by 40% between P13 and P30 in the Ca_V_1.4(α_1F_)-KO mice (average number of Bassoon puncta: 0.0057/µm^3^±0.0005 at P13 vs 0.003/µm^3^±0.0001 at P30, independent student t-test, p<0.0001). Furthermore, its association with Ribeye – marked with CtBP2 - also significantly diminished with time: 75.6% of Bassoon puncta were associated with CtBP2 at P13, whereas only 61.5% of Bassoon puncta were associated with CtBP2 at P30 (independent student t-test, p<0.05). Given that in young pups a substantial amount of Bassoon was associated with CtBP2, we hypothesized that ribbons may be anchored in young pups. Using electron microscopy, we analyzed the morphology of photoreceptor terminals of P13 and P46 Ca_V_1.4(α_1F_)-KO and age-matched WT retinae. In the latter, cone and rod terminals were readily identifiable by their size as well as the number of ribbons, invaginations and mitochondria. In Ca_V_1.4(α_1F_)-KO retinae, terminals were extremely misshapen and in most cases did not display the morphological features one commonly relies upon to differentiate cone and rod terminals. On rare occasions, in the young pups, we found identifiable cone terminals. The pedicle in figure 4J from a P13 Ca_V_1.4(α_1F_)-KO retina was identified as a cone pedicle as it displays some attributes resembling age-matched WT retinae (figure 4I). The size of the two pedicles is rather similar and ribbons, much shorter but anchored, can be seen in the Ca_V_1.4(α_1F_)-KO (white arrows – figure 4J). At P46, however, we could not determine if this terminal was a rod or cone terminal, as the usual ultrastructural features were absent (figure 4K). In line with data previously presented, the ribbons in this terminal are not anchored and adopt a spherical shape. These data are coherent with the decrease in Bassoon immunoreactivity between P13 and P30. Also, in line with data reported by Raven et al [17], we never found any invagination in Ca_V_1.4(α_1F_)-KO retinae. We further investigated the expression of Rim2, a protein known to interact with both Bassoon [26] and the CaV1.4(α_1F_) channel [51] in ribbon synapses. At all ages it co-localized with Bassoon, thus followed the same behavior (data not shown).

### Other Synaptic Proteins in the Ca_V_1.4(α_1F_)-KO

**Figure 5:**
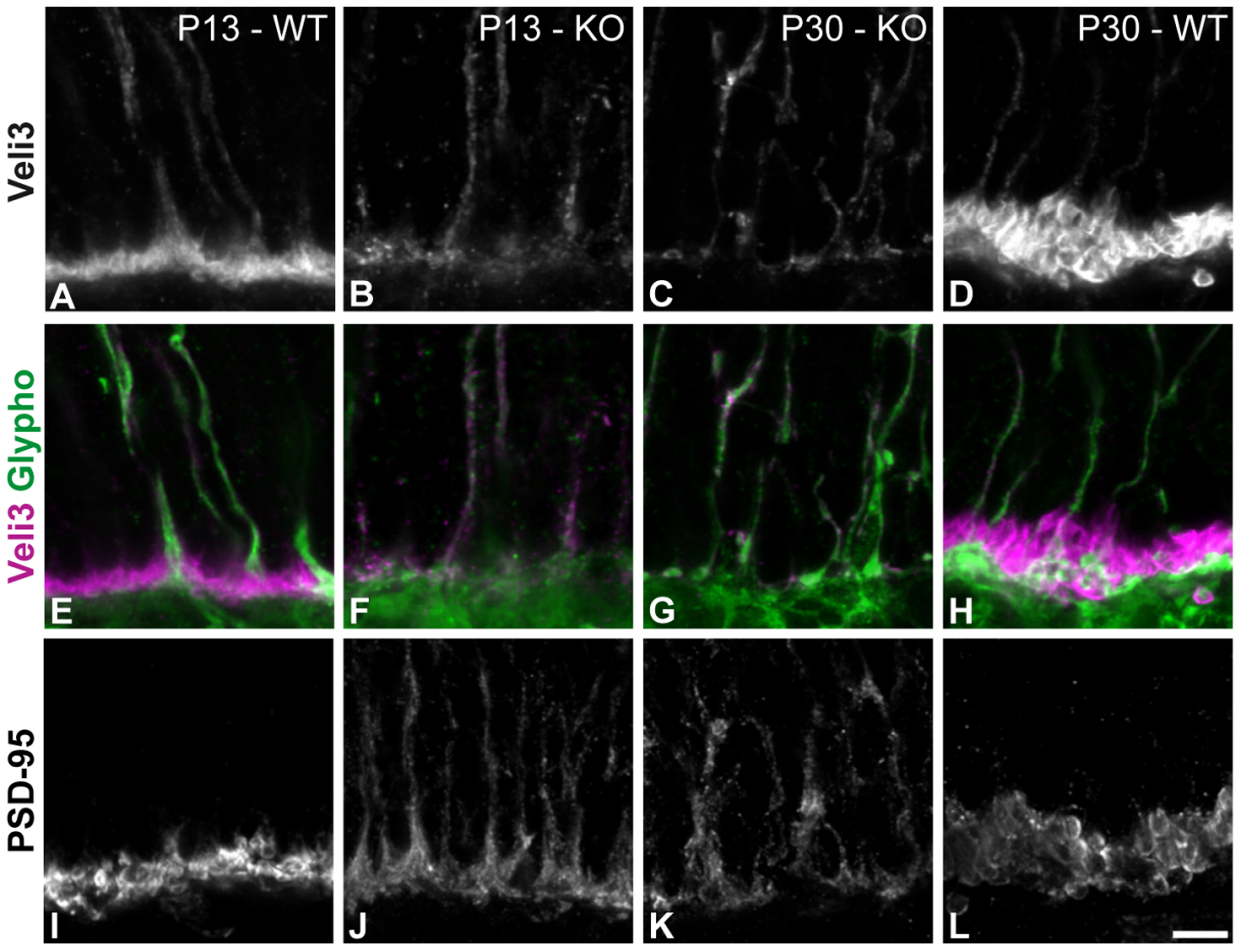
Expression of additional presynaptic proteins. (**A–L**) Vertical sections from P13-WT (**A, E and I**), P13-KO (**B, F, and J**), P30-KO (**C, G and K**) and P30-WT (**D, H and L**). The immunoreactivity of Veli3 is presented alone in the top row in grayscale (**A–D**). In the second row Veli3 (magenta) and glycogen phosphorylase (Glypho, green) signals are presented merged (**E–H**). PSD-95 immunoreactivity is shown in panels (**I–L**). Scale bar = 5 µm.

Moreover, we analyzed the expression of two additional presynaptic proteins, Veli3 and postsynaptic density-95 (PSD-95). Veli3 is an adaptor protein present in photoreceptor terminals [29]. The scaffolding protein PSD-95 is found in both pre- and postsynaptic elements, including photoreceptor terminals [30]. Figure 5 shows that neither Veli3 nor PSD-95 were normally distributed in Ca_V_1.4(α_1F_)-KO photoreceptor terminals. They displayed different patterns of expression. Veli3 expression is presented alone in the top row (figure 5A–D), and merged with Glycogen phosphorylase (Glypho), a marker of all cones [40], in the second row (figure 5E–H). Veli3 immunofluorescence was strongly down-regulated in rod photoreceptors but appeared to be maintained in cones, including in their synapses. At P13, PSD-95 was already expressed at the synapses, particularly in rod terminals, in the WT (figure 5I), while the expression in Ca_V_1.4(α_1F_)-KO was much less intense and not concentrated in the terminals at the same age (figure 5J). At P30, PSD-95 was still not localized to the terminals (figure 5K–L). Given that terminals in the OPL as well as ectopic ones were full of vesicles in Ca_V_1.4(α_1F_)-KO retinae, we set out to establish if the vesicle machinery was maintained.

### Vesicle-associated Proteins in the Ca_V_1.4(α_1F_)-KO

**Figure 6:**
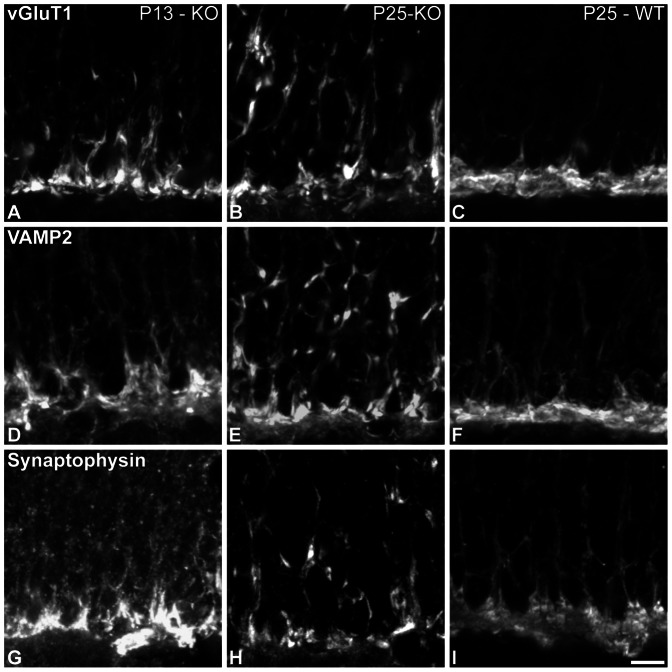
Expression of vesicular proteins. (**A–I**) Vertical sections from P13-KO (**A, D and G**), P25-KO (**B, E and H**), and P25-WT (**C, F and I**). Confocal micrographs of retinae immunolabeled for vGluT 1 (**A–C**), VAMP2. (**D–F**) and synaptophysin (**G–I**). Scale bar = 5 µm.

Vesicular glutamate transporter 1 (vGluT1) was expressed at the ectopic and OPL synapses, at all ages analyzed (figure 6A–C). VAMP2 (vesicle-associated membrane protein 2/synaptobrevin 2) is a vesicular SNARE protein. Its expression was clear and present at both ectopic and OPL terminals in the Ca_V_1.4(α_1F_)-KO retinae at all analyzed ages (figure 6D–F). Synaptophysin is an integral membrane protein of synaptic vesicles [52]. No changes were observed in this protein’s expression pattern (figure 6G–I). Furthermore, as already shown in figure 2, expression of Cplx4– a cytosolic synaptic protein involved in vesicle exocytosis – was also maintained at the synapse at all studied ages. Morphologically, all vesicle-related markers remain unaffected in the Ca_V_1.4(α_1F_)-KO retina. While our analysis does not assess the functional aspects of synaptic release, our findings suggest that synaptic calcium and Ca_V_1.4(α_1F_) channels are not necessary for the expression, targeting and maintenance of the release machinery. This is further supported by data demonstrating that protein sorting during vesicle biogenesis is not calcium dependent (see for review [53]).

Our anatomical data are in line with developmental findings suggesting that photoreceptor terminals first acquire basic synaptic properties during the first postnatal week, before becoming ribbon synapses during the second postnatal week [32] and completing their maturation after eye opening [54].

Overall these data demonstrate, that in Ca_V_1.4(α_1F_)-KO mice photoreceptor terminals are not entirely normal in young pups (Veli3 and PSD-95 are not expressed in rod terminals), but that most of the defects appear following eye opening. Moreover, in pups short synaptic ribbons were anchored, whereas only floating ribbons were observed in older animals. This is further correlated with a higher expression of Bassoon as well as its apposition to CtBP2 in young pups in comparison to adults. In contrast, the expression of vesicle-related proteins remained unchanged.

### Morphology and Distribution of Cones in the Ca_V_1.4(α_1F_)-KO Retina

Given that some previously published data demonstrated that cones adopted aberrant morphologies with several branches and varicosities consistent with new synapse formation [17], we examined the behavior of cones in the Ca_V_1.4(α_1F_)-KO retina.

**Figure 7:**
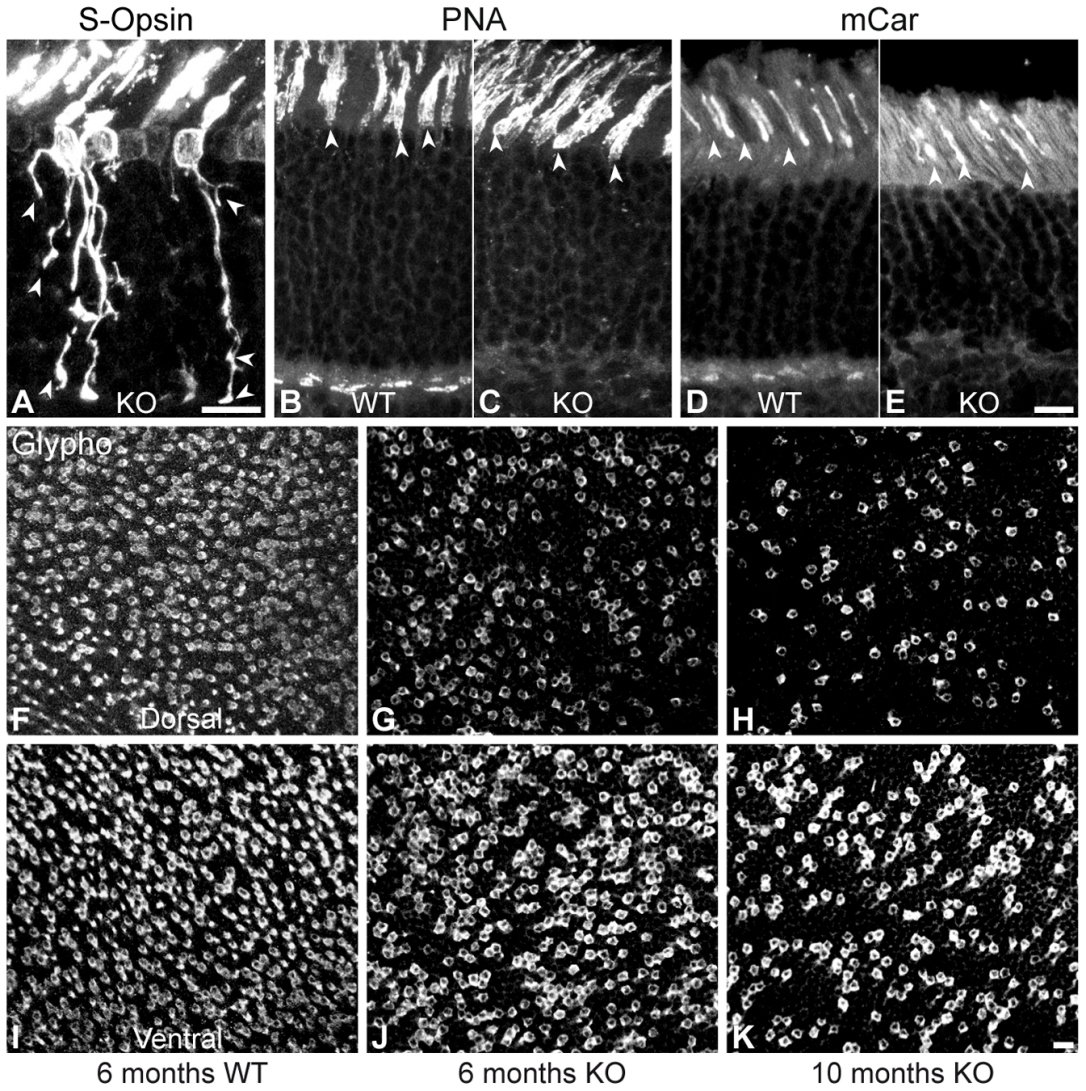
Mature cones in Ca_V_1.4_(α1F)_-KO. (**A–E**) Vertical sections from P46 Ca_V_1.4_(α1F)_-KO (**A, C, E**) and P46-WT (**B,D**). **A**: S-opsin labeling shows the morphology of cones in Ca_V_1.4_(α1F)_-KO. Arrowheads indicate the various cone branches and synapse-like terminals. The expression of two cone markers, PNA (**B–C**) and mCar (**D–E**) is presented in **B–E**. Arrowheads indicate the immunopositive outer segments of cones in both WT and Ca_V_1.4_(α1F)_-KO. (**F–K**) Glycogen phosphorylase (glypho) immunoreactivity shows the overall cone population in the dorsal (**F–H**) and ventral (**I–K**) retina of WT at 6 months (**F, I**) and of Ca_V_1.4_(α1F)_-KO at 6 months (**G, J)** and 10 months(**H, K**). Scale bars = 10 µm.

The mouse retina presents a gradient of opsin expression, where in ventral retina cones co-express M- and S-opsin, whereas pure M-opsin and S-opsin cones can be found in the dorsal retina [55]. Hence, we used S-opsin to visualize cones in Ca_V_1.4(α_1F_)-KO and WT mice as it gives a strong and graded signal, thereby allowing for better observation of individual morphologies. The S-opsin was present in the outer segments of cones at all tested ages in both WT and Ca_V_1.4(α_1F_)-KO mice, where it appeared to be normally distributed (figure 7A). In line with the evidence presented in Raven et al. [17], in some cones the S-opsin label was present across the entire cell and revealed an aberrant morphology of cones. In adult animals, (P46 – figure 7A), one could often observe very complex morphologies with several branching points and varicosities in Ca_V_1.4(α_1F_)-KO mice. In some cases, one could even discern several axons emerging from the cone soma. On occasion, the principle axon appeared retracted, although in most cases it was still attached to the OPL.

In order to ensure that cones did not lose their identity in Ca_V_1.4(α_1F_)-KO mice, we analyzed the presence and distribution of different cone markers. Peanut agglutinin (PNA) labeling demonstrated that the outer segments were still present and normal looking (figure 7B and C). However, no PNA-positive pedicles could be observed in Ca_V_1.4(α_1F_)-KO retinae. In accordance with PNA labeling, cone arrestin (mCar) immunoreactivity was present in the outer segments but not in the pedicles (figure 7D and E). M-opsin was also present in the outer segments of cones and when the entire cone was labeled, similar aberrant morphologies as in the S-opsin positive cones could be observed (data not shown).

### Cone Degeneration

Raven et al [17] showed that Ca_V_1.4(α_1F_)-KO retinae exhibit a reduced number of cones. We thus investigated the onset of cone degeneration in the Ca_V_1.4(α_1F_)-KO. We used Glycogen phosphorylase (glypho) immunoreactivity on flat-mounted retinal preparations at different ages to study cone degeneration in this model. Furthermore, in order to evaluate if all cone populations degenerate at the same rate we co-labeled retinae with S-opsin (figure 7F–K). There was no clear cone loss until 6 months of age. No obvious difference could be observed in the ventral retina (double-opsin expressing cones) of Ca_V_1.4(α_1F_)-KO when compared to the WT (figure 7I and J). However, a small decrease in the number of cones in the dorsal part of the retina was detected (figure 7F and G). We could clearly see a decrease in the number of cones over the entire retina at 10 months of age, with a greater level of degeneration in the dorsal part, corresponding to the M-opsin expressing cones (figure 7H and K).

These data confirm that cones degenerate in this model but the process is slow and heterogeneous. In addition, we demonstrate that morphological cone abnormalities can be observed several months prior to the onset of cone death. This suggests that cone reorganization could be related to plasticity rather than cell death. Thus, we investigated the onset of cone sprouting and the expression of presynaptic proteins in new cone terminals.

### Cone Sprouting and Synaptogenesis

**Figure 8:**
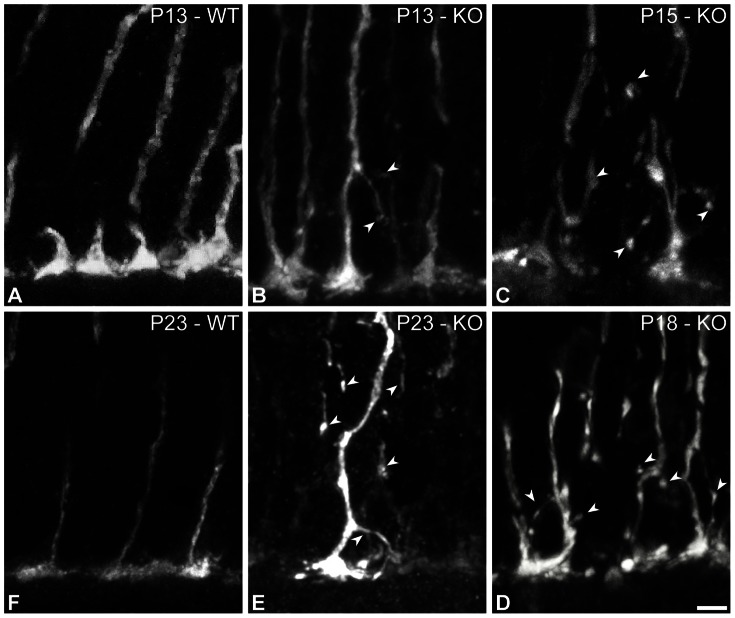
Cones in Ca_V_1.4_(α1F)_-KO at different ages. (**A–F**) Vertical sections from WT (**A, F**) and Ca_V_1.4_(α1F)_-KO (**B–E**) at different ages. S-opsin labeling shows the morphology of cones in Ca_V_1.4_(α1F)_-KO at P13 (**B**), P15 (**C**), P18 (**D**) and P23 (**E**), and in WT at P13 (**A**) and P23 (**F**). Arrowheads indicate the various cone branches and synapse-like terminals. Scale bar = 5 µm.

Figure 8 shows the axons of S-opsin expressing cones at several ages. At P13, most of the cone pedicles were smaller than age-matched WTs but their axons appeared mostly normal (figure 8A and B). One should, however, mention that some sparse examples of sprouting could be seen (arrowheads - figure 8B). Cone morphology became progressively more abnormal over the following days (figure 8C–E).

**Figure 9:**
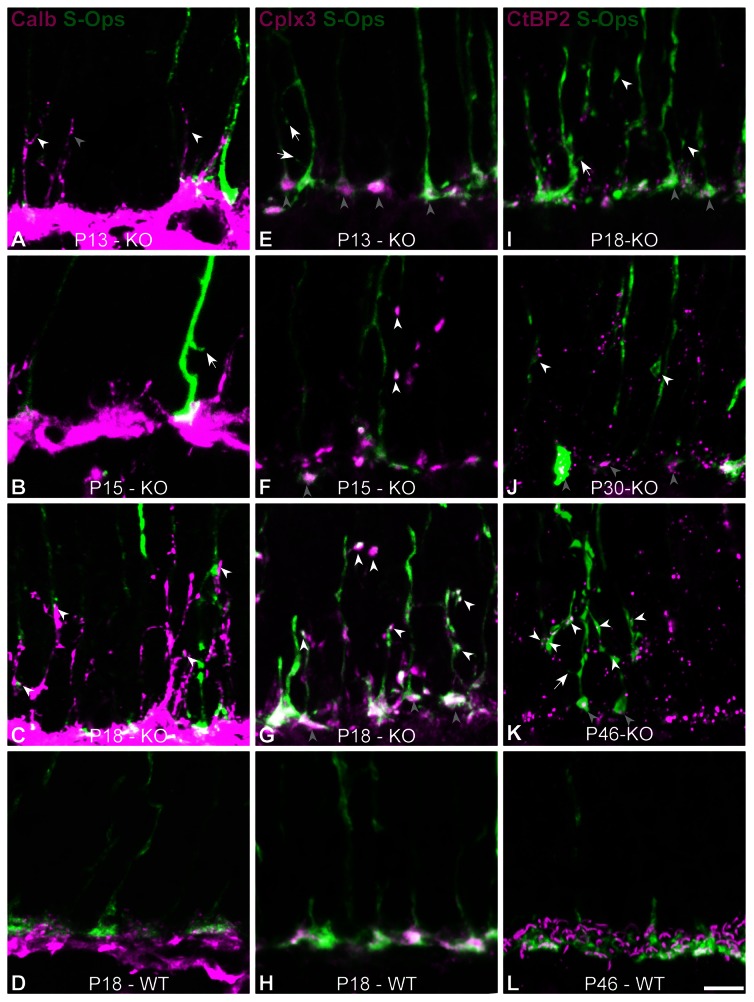
Cones in Ca_V_1.4_(α1F)_-KO establish new synapses. (**A–D**) Vertical sections from Ca_V_1.4_(α1F)_-KO at different ages (**A–C**) and WT (**D**). S-opsin (green) and calbindin (magenta) labeling show the morphology of cones in Ca_V_1.4_(α1F)_-KO and sprouting of horizontal cells at P13 (**A**), P15 (**B**), P18 (**C–D**). Arrowheads indicate the fasciculation or apposition of HC neurites onto cones and the arrow points to a cone sprout. (**E–H**) Vertical sections from Ca_V_1.4_(α1F)_-KO at different ages (**E–G**) and WT (**H**). S-opsin (green) and complexin 3 (magenta) immunoreactivity shows progressive appearance of Cplx3 in new cone synapses (white arrowheads). Grey arrowheads indicate the Cplx3-positve cone pedicles. (**I–L**) Vertical sections from Ca_V_1.4_(α1F)_-KO at different ages (**I–K**) and WT (**L**). S-opsin (green) and CtPB2 (magenta) expression shows progressive appearance of ribbons in new cone synapses (white arrowheads). Scale bar = 5 µm.

Given that horizontal cells (HCs) are known to extend neurites in the ONL of this model, we hypothesized that they were the most probable candidates to establish ectopic contacts with cones [8], [16]. Figure 9A–D shows the double labeling of HCs (calbindin) and S-opsin at different ages. At P13, cone structure is mostly normal, but HCs have already started sprouting and some fasciculate onto cone axons (figure 9A). Supporting the idea that cones seek new partners, we found clear examples of cones developing sprouts (figure 9B). Finally at P18, we observed clear ectopic appositions between cone and HC sprouts in the ONL (figure 9C).

In order to evaluate if those appositions could differentiate into synapses, we investigated the expression of some presynaptic markers at cone pedicles and ectopic terminals. Some cone markers, such as mCar and PNA, were absent from both normal and ectopic cone terminals (see figure 7). Other proteins such as complexin 3 (Cplx3) and the ribbon marker CtBP2 were expressed at both sites. At P13, synaptic proteins were expressed only in the cone pedicles (figure 9E - grey arrowheads). At P15, some ectopic terminals were Cplx3-positive (figure 9F – white arrowheads). By P18, however, a large number of ectopic terminals were Cplx3-positive (figure 9G), while in WT all cone pedicles were OPL bound and expressed Cplx3 (figure 9H). CtBP2 expression in cone ectopic synapses followed a slower timeline than Clpx3, as only very few CtBP2-positive ectopic terminals were present before P30 (figure 9I and J). At P46 however, we observed several CtBP2 puncta in ectopic cone terminals (figure 9K). It is also noteworthy that even at P46 there were small sprouts emanating from the cones (figure 9K – white arrows) suggesting that cone morphogenesis was on-going. Furthermore, these data demonstrate that cones establish ectopic contacts containing at least some elements of the synaptic machinery in the Ca_V_1.4(α_1F_)-KO retina.

Together these data demonstrate that in Ca_V_1.4(α_1F_)-KO mice photoreceptor terminals develop with only subtle anomalies, but that several scaffolding elements are not maintained following eye opening. Furthermore, cones retain developmental abilities even in adults. This suggests that the defects observed in the adult are in part due to developmental issues and in part due to lack of maturation that should occur following the onset of vision.

## Discussion

This study explores the postnatal expression of synaptic proteins and the behavior of cones in the retina of Ca_V_1.4(α_1F_)-KO mouse. Several scaffolding elements in the ribbon synapse are affected. The distribution of proteins such as Veli3 and PSD-95 is abnormal in photoreceptor terminals of young pups, while Bassoon and RIM2 are properly targeted at first but their expression and localization are not maintained in adult, indicating that either activity or the physical presence of the channel are necessary for their proper expression and localization. The defects overlap in several aspects between rods and cones, but cone synaptic protein distributions are better preserved. We also demonstrate that cones are plastic in this model. They are capable of sprouting and establishing ectopic synaptic terminals, to which at least two synaptic proteins are dispatched.

### Ribbon Synapse Development

Several classical studies have contributed to a detailed description of ribbon synapse development in the mouse retina [56]–[58]. In cone terminals, it is initiated at P4/P5 by contacting one HC dendrite and attaching the ribbon to the presynaptic membrane. Around P6, another HC dendrite is recruited and the complex invaginates into the terminal. Between P7 and P10, ON cone bipolar cell dendrites invade the terminal and assume the central position underneath the ribbon within the invagination. Rod synaptogenesis follows a similar sequence of events starting at P8. Rod bipolar cell dendrites begin to invade the rod terminals at P10. The process is completed for both rods and cones by eye opening.

In agreement with Raven et al [17], cone terminals were abnormal at the ultrastuctural level in the Ca_V_1.4(α_1F_)-KO mice. The pedicles were smaller and no invaginations were visible at any age. Given that HC dendrites invaginate at about P6 in cones, this demonstrates that there are some developmental defects in the Ca_V_1.4(α_1F_)-KO. Bipolar cell dendrites invaginate later (between P7 and 10) and require β-dystroglycan, a presynaptic extracellular protein [59]. This protein is absent from the adult Ca_V_1.4(α_1F_)-KO retina [10]. We have not verified its absence during retinal development but that would explain the lack of bipolar cell invagination. Furthermore, PNA labeling was absent in the adult as well as developing Ca_V_1.4(α_1F_)-KO retina at the pedicle base, which indicates that at least one extracellular presynaptic element is missing in the Ca_V_1.4(α_1F_)-KO retina.

### Ribbons and Active Zone Associated Proteins

In younger animals, ribbons, although abnormally short, appeared still capable of approaching and even anchoring to the membrane in a ribbon-like shape. However, in older animals, ribbons were floating and adopted a spherical shape, very reminiscent of the precursor spheres state found around P4 in cones [18], rather than the untethered rod-shaped ones found in adult Bassoon-KO mice [28]. This indicates that the inability of ribbons to maintain their anchoring to the membrane is not the only factor in their abnormal shape. Bassoon and Piccolo immunofluorence was also reduced with age (this study and [17], respectively). Moreover, Bassoon association with the ribbon marker CtBP2 was not systematic and decreased with age. A plausible explanation for this phenomenon is that the precursor spheres, pre-assembled protein aggregates including Ribeye, Bassoon and Piccolo [18], are accurately targeted to the presynaptic membrane. However, in absence of either activity or the calcium channels themselves, they are unable to stabilize their association and anchoring. In line with this hypothesis is the fact that RIM2 was also appropriately targeted and associated to the ribbon in young pups, but not in adults. Of interest is that RIM2, along with other proteins, has been reported to associate with ribbons in the second stage of synapse formation [18], supporting the idea that the initial steps of ribbon synapse formation are preserved in the Ca_V_1.4(α_1F_)-KO mice and are therefore, not activity- or Cav(α_1F_)-dependent. Bassoon is localized at the border between ribbon-associated and active zone compartments [26]. The abnormal distribution of Bassoon, CtBP2, Piccolo and RIM2 suggests that the lack of Ca^2+^ influx and/or the absence of the channel itself could alter the organization of both synaptic compartments. The effect could be direct, as it was shown that Bassoon and calcium channels physically interact at the neuromuscular gap junction [60] and are part of the same complex at the ribbon synapse in hair cells [61]. In addition, RIM2 was recently shown to directly interact with the calcium channel as well [51]. Alternatively, the effect can be indirect, as we have shown that the immunofluorence of several scaffolding proteins was reduced in the Ca_V_1.4(α_1F_)-KO mice.

The data we have collected on ribbon anchoring is from cones. It is possible that some ribbons were also anchored in rods. However, those terminals were extremely difficult to recognize with certainty because they were extremely misshapen and the occurrence of anchored ribbons was low even in cones. It is also possible that Ca_V_1.3(α_1D_), a calcium channel that could be expressed in cones [4], [5], provided enough calcium influx or structural interactions to preserve the cone terminal to some extent.

### Additional Synaptic Proteins

**Figure 10:**
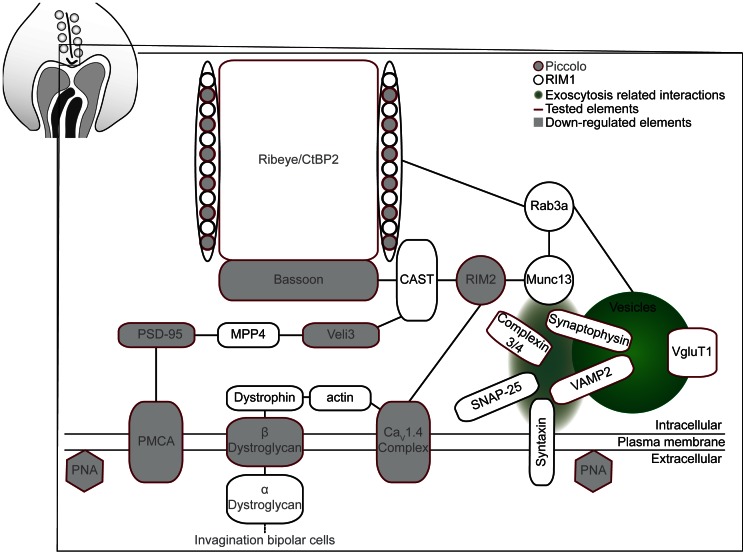
Summary of the elements affected in the photoreceptor terminals in the Ca_V_1.4_(α1F)_-KO. Several protein–protein interactions are affected such as the association of Ribeye, Piccolo and Bassoon. The investigated proteins in the model are depicted with a red outline. The proteins with an abnormal expression and/or distribution are shown in grey. Synaptic ribbons form a complex with several other proteins such as CAST, RIM2, Rab3a, and Munc13. In the Ca_V_1.4_(α1F)_-KO, RIM2 expression is compromised. The vesicle related machinery (VAMP2, synaptophysin and complexin 3 and 4) is not affected in the Ca_V_1.4_(α1F)_-KO. Other presynaptic proteins such PMCA, PSD-95 and Veli3 are also affected in the Ca_V_1.4_(α1F)_-KO. Furthermore, β-Dystroglycan, a protein necessary for the invagination of bipolar cells is absent from the photoreceptor synapses in the Ca_V_1.4_(α1F)_-KO.

In addition to Ribeye and Bassoon, we have investigated several other synaptic proteins. Figure 10 summarizes the molecular state of photoreceptor synapses from our findings and those of others in this model and similar ones. The first observation is that several membrane associated elements are missing in the adult terminals (PMCA [62], β-dystroglycan [10], PNA). Furthermore, several other intracellular synaptic proteins, such as Bassoon, Piccolo, RIM2, PSD-95 and Veli3 display an abnormal distribution and/or decreased expression in the Ca_V_1.4(α_1F_)-KO mice, while vesicular related proteins appear preserved. Although this may seem surprising given the number of calcium-dependent protein interactions of the vesicular associated proteins, one has to bear in mind that most of those interactions happen in the event of synaptic release, while the proteins affected here are important to provide the synaptic scaffold. For example, our data on the decreased RIM2 expression as well as its improper distribution in the Ca_V_1.4(α_1F_)-KO are in agreement with recently published data indicating that the level of expression and/or stability as well as the presence of RIM2 at the active zone was ribbon-dependent [51]. Of interest is the fact that a recent study demonstrated the loss of PMCA expression in the *nob2* model [62]. This finding is in line with the expression patterns of PSD-95 and Veli3 we report here, since it was demonstrated that PMCA localization is PSD-95 dependent [63]. Furthermore, the membrane-associated guanylate kinase (MAGUK) protein MPP4 has a major role in the localization, stabilization and regulation of PSD-95 protein turnover in the photoreceptor synaptic terminal [64]. It was shown that MPP4 knockout mice have similar deficits in the expression and distribution of PDS-95, Veli3 and PMCA but display only minor morphological and functional changes [64]. Thus one can further conclude that these deficits alone are not sufficient or necessary to cause rod terminal retraction in Ca_V_1.4(α_1F_)-KO.

### The Role of Calcium versus Protein Interaction with the Ca_V_1.4(α_1F_) Channel

To our knowledge no data are available on the relative importance of activity-induced calcium influx and the stabilization due to physical protein interactions in this knockout. However, several major scaffolding elements of the ribbon synapse have been knocked out [50], [65] and none produced this extent of disorganization in synaptic structure. A recent study investigated the relative importance of activity and the presence of the Cav1.3 channel in zebrafish ribbon hair cells. The authors concluded that calcium influx was necessary for the maturation of the terminal [12]. Given the similarities between their model and ours, one can suggest that activity-induced calcium influx is a vital element to stabilize the scaffolding elements in the retinal ribbon synapse.

In addition to its role in synaptic transmission, calcium is an important second messenger for several cell mechanisms. A recent study has demonstrated that calcium signals are compartmentalized with large [Ca2^+^] changes in the cone terminal but not in the soma or inner segment. [19]. However, no data are available about calcium compartmentalization during photoreceptor development or whether it is Ca_V_1.4(α_1F_)-dependent. Consequently, some of the effects we see in this model could still arise from the role of calcium as a second messenger.

### Cone Morphology in Ca_V_1.4_(α1F)_-KO

Rod plasticity has been reported in several models with synaptopathies (see for example [16], [48], [66], [67]), while cones are generally considered as relatively non-plastic. Cones have been shown to make contacts with rod bipolar cells in the absence of rods but as those contacts remained in the OPL [68], cone reorganisation was by no means as extensive as what we report in this study. To our knowledge, neurite sprouting from cone photoreceptors has never been reported in any other model of synaptopathy. Sprouting and extensive reorganisation of the cone photoreceptors was reported in one *retinitis pigmentosa* model (the rd1 mouse model), but it is not known whether cones established new synapses.

Furthermore, in the rd1 model, rod photoreceptor degeneration causes major reorganisation in the entire retinal structure [69], [70]. One similarity between the two models is that cones also degenerate. Lin et al [70] suggested that cone remodeling could be a precursor sign for cone degeneration. The data we present here seem to be in line with this hypothesis. However, sprouting and synaptogenesis happen several months prior to cell death. Moreover, even at 10 months, several cones are still present even in the dorsal retina. This suggests that even if synaptogenesis is a precursor of cell death, the latter would be an extremely slow process.

Hair cells share several attributes with photoreceptors, including the presence of ribbons. Furthermore, they express Ca_V_1.3(α_1D_), a calcium channel with very similar properties to Ca_V_1.4(α_1F_). It has been shown that prior to the onset of hearing, inner hair cells pass through a stage of strong vesicular release attributable to increased expression of Ca_V_1.3(α_1D_) channels during synaptogenesis. Toward the onset of hearing, the amount of Ca_V_1.3(α_1D_) channels declines, and efficacy of calcium induced synaptic release improves, suggesting a role for calcium in synapse maturation [71]. Our data demonstrate that the onset of cone aberrant morphology coincides with the onset of the ability of cones to respond to light (P12–13). Since electrophysiological data suggest that phototransduction occurs in Ca_V_1.4(α_1F_)-KO [8], one could hypothesize that either calcium or the insertion of the Ca_V_1.4 channel into the membrane are the trigger for proper stabilisation of the synaptic compartment and/or the maturation of the cone and its absence would allow the cone to retain some developmental abilities such as synaptogenesis.
